# Characterization of Ultrasound Probe-Dependent Interference in Electromagnetic Tracking for Image-Guided Procedures

**DOI:** 10.3390/s26134096

**Published:** 2026-06-27

**Authors:** Simão Valente, Pedro Morais, Andreas Fritz, Antonia Stern, Estêvão Lima, João L. Vilaça

**Affiliations:** 1 2Ai—Applied Artificial Intelligence Laboratory, School of Technology, Polytechnic University of Cávado and Ave (IPCA), 4750-810 Barcelos, Portugal; spvalente@ipca.pt; 2 Life and Health Sciences Research Institute (ICVS), School of Medicine, University of Minho, 4710-057 Braga, Portugal; 3 ICVS/3B’s—PT Government Associate Laboratory, 4710-057 Braga/Guimarães, Portugal; 4 LASI Intelligent Systems Associate Laboratory, 4800-058 Guimarães, Portugal; 5 Karl Storz SE & Co. KG, 78532 Tuttlingen, Germany; andreas.fritz@karlstorz.com (A.F.);; 6 Faculty of Medicine, University of Lisbon, 1649-028 Lisbon, Portugal; estevaolima@medicina.ulisboa.pt

**Keywords:** electromagnetic tracking, image-guided interventions, ultrasound technologies, minimally invasive surgery

## Abstract

Ultrasound (US) imaging is widely used to guide minimally invasive procedures such as percutaneous nephrolithotomy (PCNL), while electromagnetic (EM) tracking can complement US guidance by providing line-of-sight-independent instrument localization. However, US probes may distort the EM tracking field in a probe-dependent manner. This study characterized probe-induced EM interference for a conventional 3D/4D phased-array probe and a handheld wireless probe. Three experiments were conducted using an EM tracking system: spatial mapping of interference along each probe body, assessment of probe–sensor separation for the handheld probe, and evaluation of probe-induced tracking deviations in a simulated EM-guided PCNL setup with tracked needle and catheter sensors. EM-US calibration was then performed using low-interference sensor positions. The phased-array probe produced minimal disturbance, maintaining submillimetric positional and subdegree orientational precision across tested modes. Compared with the evaluated phased-array probe, the evaluated handheld wireless probe generated stronger, spatially localized interference, requiring ≥75 mm positional and ≥50 mm orientational separation to recover baseline precision. In the PCNL simulation, the phased-array probe maintained tracking stability, whereas the handheld probe introduced localized deviations. Both probes produced RMS calibration residuals below 1 mm under controlled conditions. These results provide device-specific baseline measurements and a workflow for probe-dependent interference assessment and sensor-placement optimization in EM-US navigation.

## 1. Introduction

Minimally invasive surgical techniques have shown increasing potential as an alternative to open surgery, revolutionizing the management of many conditions by reducing postoperative recovery time, pain, procedural risks, and morbidity [1,2]. Within urology, percutaneous nephrolithotomy (PCNL) remains the gold-standard intervention for large renal calculi and is recommended by international urological guidelines for kidney stones larger than 20 mm [3,4]. PCNL relies on a precise percutaneous puncture of the collecting system guided by fluoroscopy and/or ultrasound (US) imaging. However, this stage is technically demanding and directly dependent on the operator’s ability to visualize and reach the anatomical target, thereby influencing success and leading to complications [5,6]. For this reason, and to reduce radiation exposure during PCNL, electromagnetic (EM) tracking systems have been adopted as a guiding tool for percutaneous access due to their ability to provide real-time 3D localization without line-of-sight constraints and to their small sensor size [7]. However, because EM tracking systems do not provide anatomical context by themselves, they are commonly combined with US imaging for renal access, thereby integrating the portability, real-time feedback, and radiation-free anatomical visualization provided by US with accurate real-time localization of instruments (e.g., needles and catheters) within the US field of view (FOV) [7].

This fusion can address key limitations of US-only guidance, including dependence on maintaining the instrument within the US imaging plane, limited visualization of the complete three-dimensional instrument trajectory, and strong operator dependence, while broadening applicability across a variety of interventional procedures. Huber et al. presented a first experience based on an EM tracking system, imaging assisted by fluoroscopy or US, during needle placement [8]. Li et al. used a US platform (SonixGPS) with an EM-tracked needle, reporting reduced puncture attempts, puncture time, and length of stay versus conventional US guidance, even in complex stones [9,10]. More recently, Rodrigues et al. presented a novel real-time EM navigation system that needs US scans to verify safety along the renal puncture tract, combining three-dimensional instrument tracking with real-time anatomical verification [11,12]. Despite the good results of these systems, EM tracking systems can be affected by the presence of the US probes, which, depending on the US system and US technology, can significantly reduce tracking performance. In parallel, there has been a growing interest in the use of portable and more compact devices such as the recent handheld and wireless US probes, which may simplify system integration, reduce setup complexity and cost, maintain adequate image quality, and decrease the space occupied in the operating room [13], reflecting a broader trend toward ultrasound miniaturization, including integrated system-on-a-chip architectures and emerging wearable ultrasound platforms [14,15]. These characteristics make handheld probes particularly attractive for integration into intuitive and streamlined EM-tracked navigation workflows. However, for EM-US navigation, spatial localization of the US image plane remains necessary to register the image with tracked instruments and enable image-guided navigation.

A critical technical challenge is that EM tracking performance is highly sensitive to EM interference from surrounding equipment and conductors, particularly from the US probe. Such interference can increase positional and orientational variability, introduce localization deviations, and compromise the spatial correspondence between tracked instruments and US images, potentially affecting procedural safety. While several studies have analyzed global accuracy degradation due to operating room hardware, fewer have examined the localized interference induced by the US probe itself. Hastenteufel et al. assessed three state-of-the-art EM tracking systems working with different 3D US probes [16], while Bonmati et al. focused on interference produced by an endoscopic US probe in an EM tracking system [17]. Fonseca et al. assessed the influence of a two-dimensional (2D) US probe, a four-dimensional (4D) mechanical US probe, an analogic flexible ureterorenoscope, and a digital flexible ureterorenoscope in a surgical environment in two EM tracking systems [18]. Other works explored mitigation strategies, Sadjadi et al. introduced a real-time dynamic field distortion compensation method using redundant EM sensors to reduce field distortions [19], and Cavaliere et al. studied and compensated the EM interference generated by 2D US probes using a pre-trained witness sensor distortion model [20]. Pham et al. studied the optimum configuration, including the distance between the EM tracking system and the sensor and the probe–sensor distance, to provide maximum accuracy [21]. Together, these works demonstrate that US probes can be a significant source of EM distortion and that several mitigation approaches have been proposed.

Although previous studies evaluated global EM tracking degradation in surgical environments and assessed specific interactions between EM trackers and medical devices, none systematically mapped the spatial distribution of the interference generated specifically by US probe bodies, nor established probe-dependent low-interference sensor-placement regions relevant for procedures such as PCNL. Moreover, the growing adoption of compact handheld wireless probes introduces additional electronic components and potential EM noise sources that are not yet fully characterized. Addressing these gaps is essential for the reliable fusion of EM tracking and US imaging in PCNL and other image-guided interventions.

Building on previous studies of US-induced EM interference, this work provides an integrated, probe-specific evaluation framework for two US probes with distinct architectures and defines practical guidelines for their integration with EM tracking. Specifically, we: (i) perform dense spatial mapping of the interference field around each probe; (ii) determine the probe-specific distance threshold at which an EM sensor can be placed without degrading positional or orientational precision; (iii) evaluate the effect of the probes in a simulated PCNL scenario; and (iv) demonstrate the feasibility of performing EM-US calibration under the optimized sensor placement conditions. Together, these analyses provide device-specific baseline measurements and a transferable experimental workflow for evaluating US probe-induced interference in hybrid EM-US navigation systems aimed at interventions such as PCNL.

This paper is organized as follows. In Section 2, we present the experimental setup, including the EM tracking system, EM sensors, US probes, and the experimental protocols used for spatial interference mapping, distance analysis, PCNL simulation, and calibration. In Section 3 we present the results of each experiment and calibration. In Section 4, we discuss the findings and highlight the technical implications. Finally, in Section 5, we present the main conclusions of the paper.

## 2. Materials and Methods

**Figure 1:**
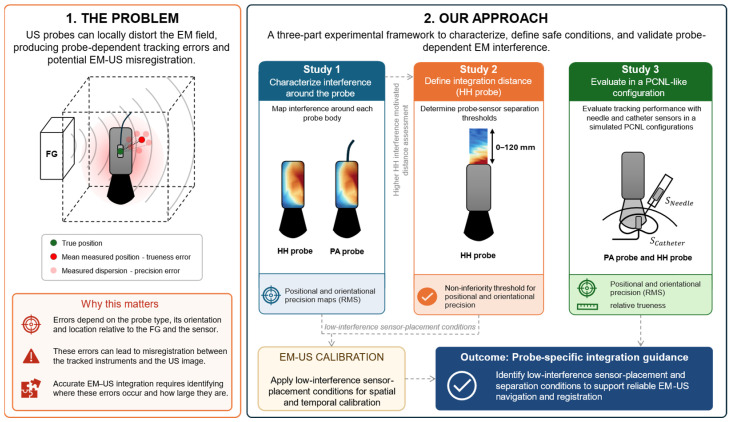
Overview of the study rationale and experimental framework for characterizing probe-induced EM interference and guiding EM–US integration. PA probe (3D/4D phased-array probe); HH probe (handheld wireless probe); $S_{Needle}$ (needle EM sensor); $S_{Catheter}$ (catheter EM sensor).

Figure 1 summarizes the rationale and sequential design of the study. Reliable EM–US navigation requires accurate spatial correspondence between tracked instruments and US images. However, probe-induced distortion of the EM field may compromise this relationship depending on probe architecture, operating state, and probe–sensor geometry. Study 1 (Section 2.3.3) therefore characterized the spatial distribution of interference around the selected handheld wireless and phased-array probes and distinguished the effects of passive probe presence from those associated with active operation. Building on these observations, Study 2 (Section 2.3.4) quantified the recovery of tracking precision with increasing separation from the handheld wireless probe and established device-specific positional and orientational distance thresholds. Study 3 (Section 2.3.5) then assessed whether the identified interference patterns remained relevant in a procedure-oriented PCNL configuration involving tracked needle and catheter sensors. Finally, EM–US calibration (Section 2.3.6) was performed under the experimentally identified low-interference conditions, linking probe characterization and sensor-placement optimization to practical system integration. The common hardware platform and experiment-specific configurations are described in the following sections.

### 2.1. Ultrasound Systems and Electromagnetic Tracking Sensors

**Figure 2:**
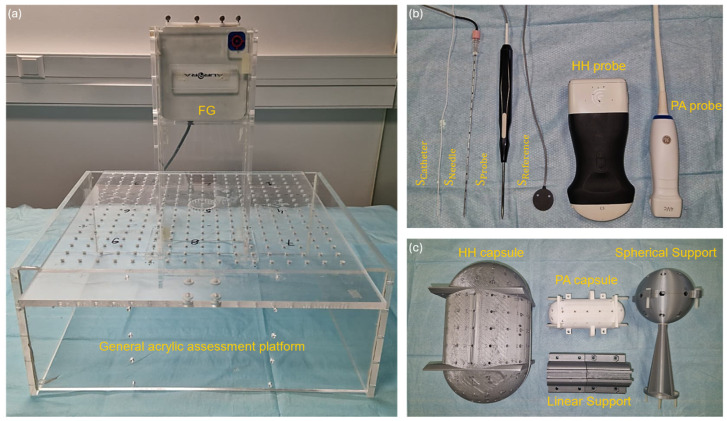
Experimental setup and equipment used for this study. (**a**) General acrylic assessment platform equipped with the Aurora field generator mounted on top, providing the basis for all experiments. (**b**) Electromagnetic (EM) sensors and ultrasound (US) probes: catheter sensor ($S_{Catheter}$, 5-DOF), needle sensor ($S_{Needle}$, 5-DOF), probe sensor ($S_{Probe}$, 6-DOF), reference sensor ($S_{Reference}$, 6-DOF), handheld US probe (Clarius C3 HD Scanner), and a 3D/4D PA probe (GE 4VC-D). (**c**) Custom 3D-printed probe support structures: HH capsule and PA capsule for Study 1, Linear support for Study 2, and spherical support for Study 3.

In this study, we evaluated the EM interference induced by two different US technologies on the NDI electromagnetic system (Northern Digital, Inc., Waterloo, ON, Canada), hereafter referred to as EMTS, using the Aurora V2 Planar 20-20 Field Generator, hereafter referred to as the FG (Figure 2a). The two US systems assessed were (Figure 2b): the Clarius C3 HD Scanner (Clarius Mobile Health Corp., Vancouver, BC, Canada), a handheld 2D US device (hereafter referred to as the HH probe), and the 4VC-D probe (GE Healthcare, Chicago, IL, USA), an active-matrix 3D/4D phased-array US transducer without internal mechanical scanning components (hereafter referred to as the PA probe), used with a Vivid E95 US system.

The FG produces a cubic EM tracking volume of approximately 500×500×500 mm, starting at approximately 50 mm from the generator surface, with submillimeter positional and subdegree orientational accuracy, which may vary depending on the sensor type used, i.e., 5 or 6 degrees of freedom (DOF) [22].

The EM sensors used in this study were: (1) the Aurora^®^ 6DOF Probe, a general-purpose digitalizing probe with a rigid metallic tip (0.3 mm diameter, 65 mm length), henceforward called $S_{Probe}$; (2) the Aurora^®^ 5-DOF Flex Tube (1.0 mm diameter), henceforward called $S_{Catheter}$; (3) the Aurora^®^ 5-DOF Needle (18G/200 mm, Chiba) with an integrated sensor in its tip, consisting of a stylet and an outer cannula, henceforward called $S_{Needle}$; and (4) the Aurora^®^ 6-DOF Reference (25 mm diameter, 5 mm thick), a flat reference disc designed for multiple fixation methods, henceforward called $S_{Reference}$ (Figure 2b).

**Figure 3:**
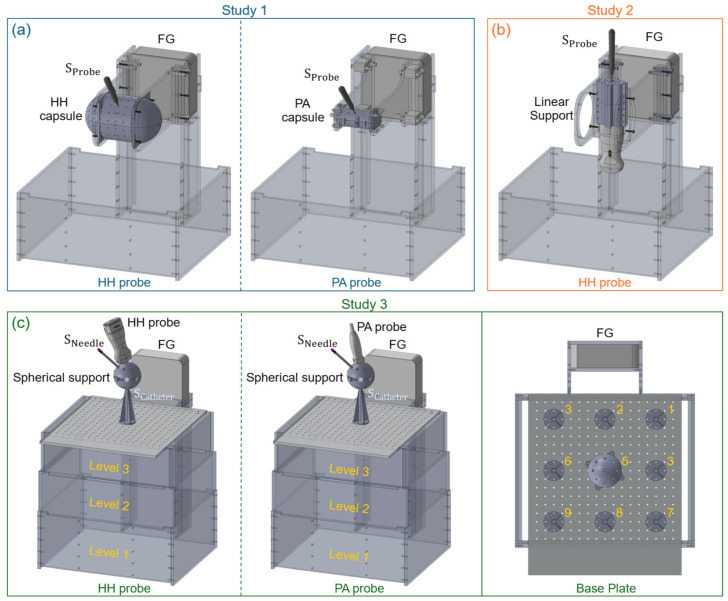
Schematic representation of the experimental configurations for the three studies. (**a**) Study 1: setup for the HH probe (left) and PA probe (right), with $S_{Probe}$ inserted into one of the holes of the HH probe (*n* = 218) and PA probe (*n* = 57) capsules, respectively. (**b**) Study 2: configuration used exclusively for the HH probe, where $S_{Probe}$ was incrementally displaced along the linear support (21 holes, 25 distances). (**c**) Study 3: setup for the HH probe (left) and PA probe (center) using the spherical support, with catheter sensor ($S_{Catheter}$) placed inside the spherical support and the needle sensor ($S_{Needle}$) inserted into one of the 25 holes of the spherical support). The right image shows a top view of the base plate illustrating the nine spatial positions per level (Levels 1–3).

Each US probe and EM sensor was used in specific experimental configurations according to the objectives of the subsequent studies, as illustrated in Figure 3 and described in Section 2.3.

### 2.2. Assessment Platform

Inspired by the work of Fonseca et al., a general acrylic assessment platform was developed to serve as the structural base for all experiments (Figure 2a) [18]. This platform allowed the FG to be fixed in a defined position and enabled modular adaptations for each specific experimental setup.

The assessment platform and its experiment-specific adaptations (see Figure 3) were designed using CAD software (SolidWorks 2025, Dassault Systèmes, SolidWorks Corporation, Waltham, MA, USA). All acrylic parts of the general platform and experimental-specific modules were 10 mm thick and were laser-cut using a GN640 laser cutting machine (Gbos Laser Technology Company, Dongguan City, Guangdong Province, China) with a cutting precision of ±0.04 mm.

Additional components that were not made of acrylic were 3D printed in polylactic acid (PLA) using an Ultimaker II Plus printer (Ultimaker B.V., Geldermalsen, The Netherlands). This printer employs the fused deposition modeling (FDM) technique, with a printing resolution of ±0.15 mm. This reported printing resolution refers to the nominal printer specification and was not used as verified dimensional accuracy of the final printed components. The printed supports were used only as positioning fixtures, not as dimensionally calibrated references. Because precision metrics were computed from repeated samples at fixed locations and relative trueness from paired Empty and US-probe measurements acquired consecutively at the same fixture location without sensor repositioning, both analyses were independent of absolute fixture dimensional accuracy.

Whenever necessary, plastic screws and fittings were used to secure components while minimizing the influence of ferromagnetic materials on the EMTS.

Specific modular adaptations of this platform for each study are illustrated in Figure 3 and described in Section 2.3.

### 2.3. Experiments

#### 2.3.1. Experimental Setup

All the experiments were conducted at 2Ai—Applied Artificial Intelligence Laboratory (School of Technology, Polytechnic University of Cávado and Ave, Barcelos, Portugal), under controlled environmental conditions and in the absence of external EM interference. The FG was fixed to the acrylic assessment platform and placed on a wooden table to minimize potential ferromagnetic effects. All experiments were performed with the platform at the same position to ensure consistent results across trials.

#### 2.3.2. Data Acquisition

A custom software application developed in MATLAB^®^ (version R2024b, The MathWorks Inc., Natick, MA, USA) was specifically implemented to acquire the position and orientation of each EM sensor and to configure the initial settings of the EMTS. For every acquisition point, 50 consecutive samples were recorded, corresponding to approximately 1.25 s of continuous tracking at an update rate of 40 Hz (tracking frequency). This acquisition length was selected to provide a consistent estimate of short-term tracking variability under static conditions while maintaining a practical acquisition time across the large number of measurement points and experimental configurations. Each record contained the 3D positional data (x, y, z) and orientation in quaternions ($q_{0}$, $q_{x}$, $q_{y}$, $q_{z}$). Across all studies, in the active US-probe configurations, the probes were operated while actively transmitting acoustic energy and generating live images during EM data acquisition, rather than being merely powered on in a static non-imaging state.

#### 2.3.3. Study 1—Spatial Characterization of US Probe-Induced Interference

The aim of this experiment was to quantify the spatial distribution of EM interference along the bodies of both US probes. This analysis aimed to identify regions of maximum and minimum EM interference around each transducer.

Two perforated capsules (Figure 3a), each replicating the external geometry of the PA probe and HH probe (hereafter referred to as the PA capsule and HH capsule, respectively), were developed to accurately position the $S_{Probe}$ at predefined locations around each US probe. The HH capsule contained 218 holes separated at 30°, while the PA capsule included 57 holes separated by 45°. In both cases, each hole had a diameter of 3.1 mm, ensuring repeatable sensor alignment relative to the probe surface. The capsules were mounted at the center of the acrylic assessment platform (Figure 3a), coinciding with the FG working volume. For each measurement location, the same physical hole was used across the corresponding Empty, Off, and active-probe configurations.

**Figure 4:**
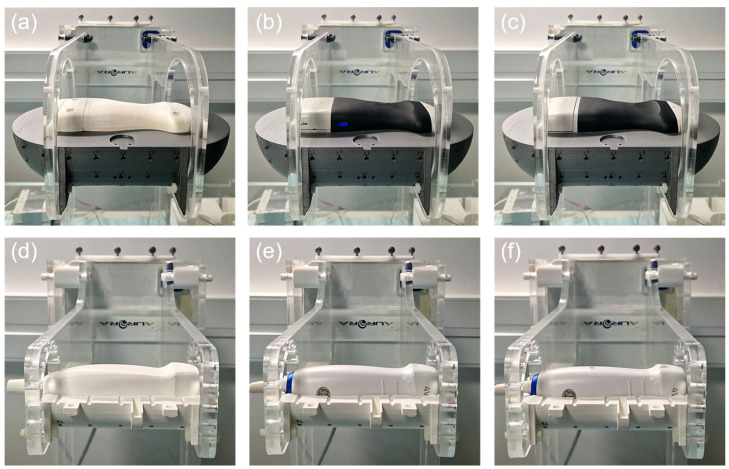
Experimental setup for Study 1—Spatial characterization of US probe-induced interference. (**a**–**c**) HH probe mounted inside the perforated HH capsule: (**a**) Empty, (**b**) Battery-Down, and (**c**) Battery-Up. (**d**–**f**) PA probe mounted inside the PA capsule: (**d**) Empty, (**e**) 2D mode and 4D mode, (**f**) 2D-Rotated and 4D-Rotated. The inactive (Off) condition used the same physical setup as the corresponding probe-present configuration and differed only in the operating state.

A 3D-printed PLA mock-up of each probe was used to acquire baseline (Empty) measurements, replicating the probe-specific geometry, ensuring that $S_{Probe}$ was positioned in the same location in both Empty and US probe configurations. For the HH probe, data were collected under four configurations: (i) Empty, (ii) Off (HH probe powered off with the battery inserted), (iii) Battery-Down (HH probe active, battery facing downward), and (iv) Battery-Up (HH probe active, battery facing upward). For the PA probe, six configurations were tested: (i) Empty, (ii) Off (PA probe physically present with the US system powered off), (iii) 2D mode (standard B-mode imaging), (iv) 4D mode (real-time 3D/4D volumetric imaging), (v) 2D-Rotated (2D mode rotated 180°), and (vi) 4D-Rotated (4D mode rotated 180°). The physical setups for the HH probe and PA probe configurations are illustrated in Figure 4.

These configurations were designed to distinguish baseline tracking variability (Empty), the passive effect of the physical probe (Off), and the aggregate effect of active probe operation (active configurations). For the PA probe, the 2D and 4D configurations additionally allowed the effect of imaging mode to be assessed. For the active configurations of both probes, data were acquired in two orientations rotated 180° from each other, simulating the complete-probe reorientation routinely performed by clinicians and allowing assessment of whether the interference pattern depended on probe orientation relative to the FG and tracked sensor. The protocol was designed to evaluate clinically relevant operating states and complete-probe orientations rather than isolate the contributions of individual internal electronic subsystems. In all cases, 50 samples per position were recorded for subsequent positional and orientational precision analysis, as described in Section 2.4.

#### 2.3.4. Study 2—Effect of Probe–Sensor Separation for the Handheld US Probe

This study aimed to determine the probe-specific separation threshold at which the EM sensor could be positioned relative to the HH probe while preserving tracking precision and maintaining practical usability. This analysis was performed only for the HH probe because its handheld design can be more susceptible to short-range EM interference, and preliminary observations from Study 1 indicated stronger distortion near this probe.

A custom perforated support (hereafter referred to as Linear support) was designed and 3D-printed to hold the HH probe and align the $S_{Probe}$ at controlled distances from the probe surface. The support featured a two-level matrix of 3×7 holes (3.1 mm diameter, 8 mm spacing), which constrained the lateral position and orientation of the sensor relative to the HH probe. The probe–sensor separation along the probe axis was manually adjusted from 0 to 120 mm in nominal 5 mm increments using a ruler marked on the probe. The Linear support was mounted at the center of the acrylic assessment platform, ensuring geometry alignment with the FG (see Figure 3b).

Measurements were acquired under two configurations: (i) Empty and (ii) Active (HH probe turned on). The data acquisition was performed using the same MATLAB^®^ software as in Study 1, collecting positional and orientational data from 525 measurement points (21 holes × 25 distances). Positional and orientational precision was evaluated at each measurement point as described in Section 2.4.

#### 2.3.5. Study 3—Effect of US Probes During Simulated PCNL

This experiment aimed to evaluate the EM interference generated by the US probes in the EMTS during a simulated PCNL procedure, where both the $S_{Catheter}$ and $S_{Needle}$ were positioned inside the kidney-mimicking setup.

A perforated spherical support (hereafter referred to as Spherical support) with a 50 mm radius was designed to reproduce the spatial arrangement of a renal cavity, enabling repeatable placement of the $S_{Catheter}$ and $S_{Needle}$ in predefined positions and orientations. The Spherical support, designed in CAD and 3D-printed under the same conditions as in previous studies, featured hexagonal holes spaced 45° apart and was mounted on a multi-level acrylic structure consisting of a perforated base plate (17×17 holes, 25 mm spacing) and three height levels separated by 150 mm (Figure 3c). The same setup was used for both the HH probe and the PA probe, each positioned adjacent to the needle trajectory to maintain the needle within the US imaging plane, reproducing the typical alignment used in PCNL. For measurement holes facing the FG, this arrangement positioned the US probe between the FG and the tracked sensors. The same nominal measurement positions were used across all tested configurations.

Measurements were acquired under four configurations: (i) Empty, (ii) PA2D (PA probe in 2D mode), (iii) PA4D (PA probe in 4D mode), and (iv) HH (HH probe turned on). Data acquisition was performed using the same MATLAB^®^ software as in the previous studies, recording positional and orientational data from both sensors at 675 measurement points (25 holes per position × 9 positions × 3 height levels).

Positional and orientational precision and trueness were computed for each configuration as described in Section 2.4.

#### 2.3.6. EM-US Calibration

Finally, an EM-US calibration was performed to estimate the spatial and temporal relationship between the US image plane and the EM sensor coordinates under the low-interference sensor-placement conditions identified in the previous studies.

**Figure 5:**
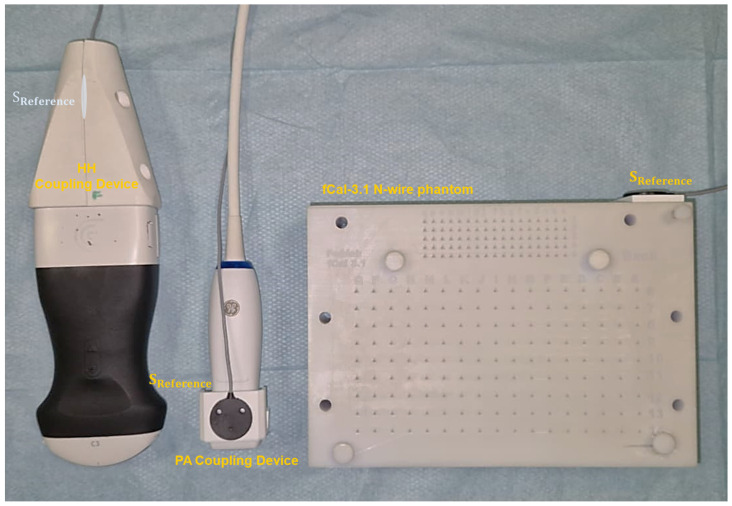
Experimental setup used for EM-US calibration. HH and PA probes equipped with their respective coupling devices and attached reference sensors ($S_{Reference}$).

A phantom-based N-wire calibration method was employed following the approach described by Carbajal et al. [23]. The fCal-3.1 N-wire phantom from the Plus toolkit was 3D-printed in TuskXC2700W resin and incorporated nine nylon wires (diameter = 0.1 mm) arranged in a 3D grid to enhance spatial calibration for deep imaging (Figure 5) [24]. Three EM sensors were used: the $S_{Probe}$ to register the phantom geometry, a $S_{Reference}$ fixed to the phantom to define its coordinate frame, and another $S_{Reference}$ attached to each US probe according to the optimal sensor placement determined from Studies 1 and 2. The HH probe operated with its default configuration (22 fps, 70° FOV, 15 cm depth, and 640×480 pixel resolution, pixel size = 0.362 × 0.362 mm), while the PA probe operated at 63 fps, 90° FOV, 16 cm depth, and 1024 × 721 pixel resolution (pixel size = 0.222 × 0.222 mm). Custom C++ software was developed to establish real-time communication between the US systems and the fCal module of the PLUS Toolkit (version 2.8.0) via the OpenIGTLink protocol. The application integrated directly with the Clarius CAST API and the GE AppAPI, allowing simultaneous acquisition of US image streams and EM tracking data for calibration.

The calibration procedure included: (i) Phantom registration, performed by collecting multiple tracked points on N-wire intersections using the $S_{Probe}$, establishing the transformation between the phantom and EMTS; (ii) Temporal calibration, estimating the time offset between the EMTS and US imaging system through an up-down probe motion over a planar surface, following the method described by Lasso et al. [25]; and (iii) Spatial calibration, matching the visible N-wire intersections in the US image with their tracked 3D coordinates to compute the rigid transformation matrix between the EMTS and US coordinate frames. Calibration parameters were obtained with a closed-form least-squares solution, minimizing the in-plane error across the imaged area [26]. The calibration error reported by the fCal module was used as an RMS calibration residual, reflecting the internal fitting consistency of the estimated EM-US calibration transformation.

Each calibration was repeated ten times for both probes, and RMS calibration residuals were reported as mean ± standard deviation.

### 2.4. Metrics

The EM tracking data were analyzed to evaluate positional and orientational precision for each experimental configuration. The precision metrics represent the local stability (jitter) of the sensor within the EM field. In Study 3, positional and orientational relative trueness were additionally assessed by comparing the active US-probe conditions with the corresponding Empty condition.

The positional precision (${Pre}_{p}$) for each location i was defined as the root-mean-square Euclidean deviation of the positional samples $P_{ij}$ from their mean position:

(1) $${Pre}_{p}(i)=\sqrt{\frac{1}{N_{s}}\sum_{j=1}^{N_{s}}{\left\|p_{ij}-{\bar{p}}_{i}\right\|}_{2}^{2}}$$

where ${\bar{p}}_{i}=\frac{1}{N_{s}}\sum_{j=1}^{N_{s}}p_{ij}$, $p_{ij}\in R^{3}$, $i=1,\ldots ,N_{L}$, j = 1, …, $N_{S}$. Here, $N_{L}$ represents the number of measurement locations (holes or positions) and $N_{S}=50$ is the number of samples per location.

For Studies 1 and 2, the orientational precision for each location i was calculated from the complete three-dimensional orientation provided by the 6-DOF sensor and was defined as:

(2) $${Pre}_{o}\left(i\right)=\sqrt{\frac{1}{N_{s}}\sum_{j=1}^{N_{s}}\;{\left(2\arccos\left(\left|q_{ij}^{T}\;{\bar{q}}_{i}\right|\right)\right)}^{2}}$$

where ${\bar{q}}_{i}$ is the unit eigenvector associated with the largest eigenvalue of $\sum_{j=1}^{N_{s}}q_{ij}q_{ij}^{T}$, $q_{ij}\in H$ is the normalized quaternion corresponding to sample j, with $i=1,\ldots ,N_{L}$, and j = 1, …, $N_{S}$. The superscript T denotes transposition, and the absolute value accounts for the fact that q and −q represent the same physical orientation.

In Study 3, the catheter and needle were tracked using 5-DOF sensors, which provide the direction of their longitudinal axis but not rotation about that axis. Therefore, orientational precision for each location i was defined from the longitudinal-axis direction as:

(3) $${Pre}_{o}\left(i\right)=\sqrt{\frac{1}{N_{s}}\sum_{j=1}^{N_{s}}\;{\left(\arccos\left(u_{ij}^{T}\;{\bar{u}}_{i}\right)\right)}^{2}}$$

where $u_{ij}$ is the unit longitudinal-axis direction obtained from quaternion $q_{ij}$, and ${\bar{u}}_{i}=\frac{\frac{1}{N_{s}}\sum_{j=1}^{N_{s}}\;u_{ij}}{{\left\|\frac{1}{N_{s}}\sum_{j=1}^{N_{s}}\;u_{ij}\right\|}_{2}}$ is the normalized mean longitudinal-axis direction at location i. This formulation excludes rotation about the longitudinal axis, which is not measured by the 5-DOF sensors.

In Study 3, relative trueness was assessed by comparing measurements acquired with the active modes against those obtained under Empty conditions. The Empty configuration served as a relative reference condition.

The positional relative trueness for each location i was defined as the Euclidean distance between the mean positions of the two configurations as

(4) $${Tru}_{p}(i)={\left\|{\bar{p}}_{empty,i}-{\bar{p}}_{US,i}\right\|}_{2}$$

where ${\bar{p}}_{empty,i}=\frac{1}{N_{s}}\sum_{j=1}^{N_{s}}p_{empty,ij}$, ${\bar{p}}_{US,i}=\frac{1}{N_{s}}\sum_{j=1}^{N_{s}}p_{US,ij}$, $p_{ij}\in R^{3}$, $i=1,\ldots ,N_{L}$, j = 1, …, $N_{S}$. This metric expresses the mean positional displacement of the tracked position relative to the Empty condition, associated with the presence and operation of the US probe.

The orientational relative trueness was similarly computed as the angular difference between the mean longitudinal-axis directions of the two configurations as

(5) $${Tru}_{o}\left(i\right)=arccos\left({\bar{u}}_{empty,i}^{T}{\bar{u}}_{US,i}\right)$$

where ${\bar{u}}_{empty,i}$ and ${\bar{u}}_{US,i}$ are the normalized mean longitudinal-axis directions for the Empty and active US-probe conditions, respectively. This formulation quantifies the measured orientation change associated with probe presence and operation while excluding rotation about the longitudinal axis, which is not measured by the 5-DOF sensors.

The RMS formulation was selected as the primary precision measure because it summarizes sample dispersion around the mean position or orientation as a single value in the corresponding measurement units while giving greater weight to larger deviations that may affect tracking stability, consistent with previous EM tracking assessments involving US probes and image-guided procedures [17,18,21,27]. To provide complementary information on the upper tail and maximum observed tracking variability, the 95th percentile (P95) and maximum sample-wise positional and orientational deviations were calculated at each measurement location in Studies 1–3. These metrics were calculated from the same Euclidean or orientational deviations used in the corresponding RMS precision calculation and are reported in Supplementary Material S4 (Tables S1–S3).

### 2.5. Statistical Analysis

Statistical analyses were performed in MATLAB^®^. All metrics are reported as median and interquartile range (IQR), and statistical significance was set at *p* < 0.05.

In Study 1, differences in positional and orientational precision among the multiple experimental conditions were assessed using Friedman tests for repeated measures, followed by pairwise post hoc Wilcoxon signed-rank tests with Bonferroni correction.

In Study 2, a one-sided Wilcoxon signed-rank non-inferiority test was applied to assess whether precision values obtained at each distance were non-inferior to those of the Empty configuration within the predefined margin. The non-inferiority margin (δ) for positional and orientational precision was derived separately from the 525 Empty-condition precision values as the largest value retained within the interval $\left[Q_{1}-kIQR,\;Q_{3}+kIQR\right]$. A study-specific factor of k = 1.25 was empirically selected as a more restrictive alternative to the conventional k = 1.50 Tukey fence [28], yielding smaller and therefore more conservative non-inferiority margins, in line with adjusted-boxplot methods that adapt IQR-based fences for skewed distributions [29].

In Study 3, inter-system differences were analyzed using Friedman test for repeated measures, followed by Bonferroni-corrected Wilcoxon signed-rank post hoc tests to identify pairwise differences. Kendall’s W quantified agreement across systems, and effect sizes (r) were computed for pairwise contrasts. For the trueness analysis, one-sided Wilcoxon signed-rank tests were applied to assess whether positional and orientational errors exceeded EMTS manufacturer’s specification (0.70 mm and 0.20°, respectively) [22].

## 3. Results

### 3.1. Study 1—Spatial Characterization of US Probe-Induced Interference

**Figure 6:**
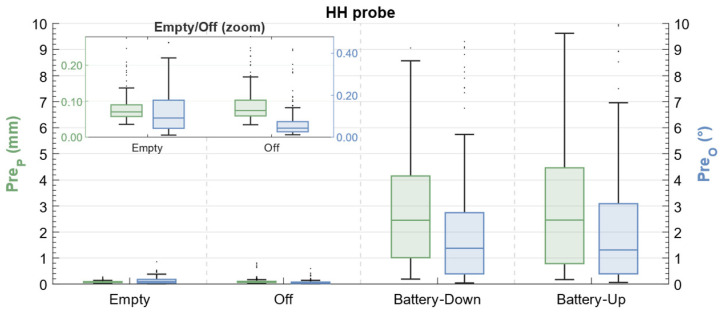
Positional and orientational precision of the EMTS under the HH probe configurations. Boxplots show positional precision in mm and orientational precision in degrees for Empty, Off, Battery-Down, and Battery-Up. Insets show zoomed views of the Empty and Off conditions.

**Figure 7:**
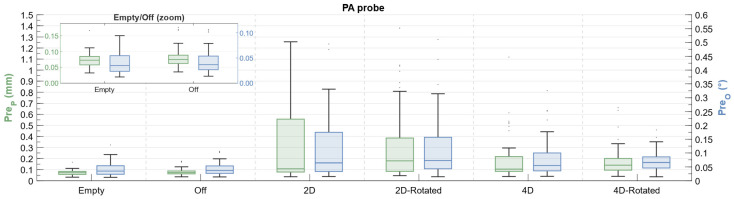
Positional and orientational precision of the EMTS under the PA probe configurations. Boxplots show positional precision in mm and orientational precision in degrees for Empty, Off, 2D, 4D, 2D-Rotated, and 4D-Rotated. Insets show zoomed views of the Empty and Off conditions.

**Figure 8:**
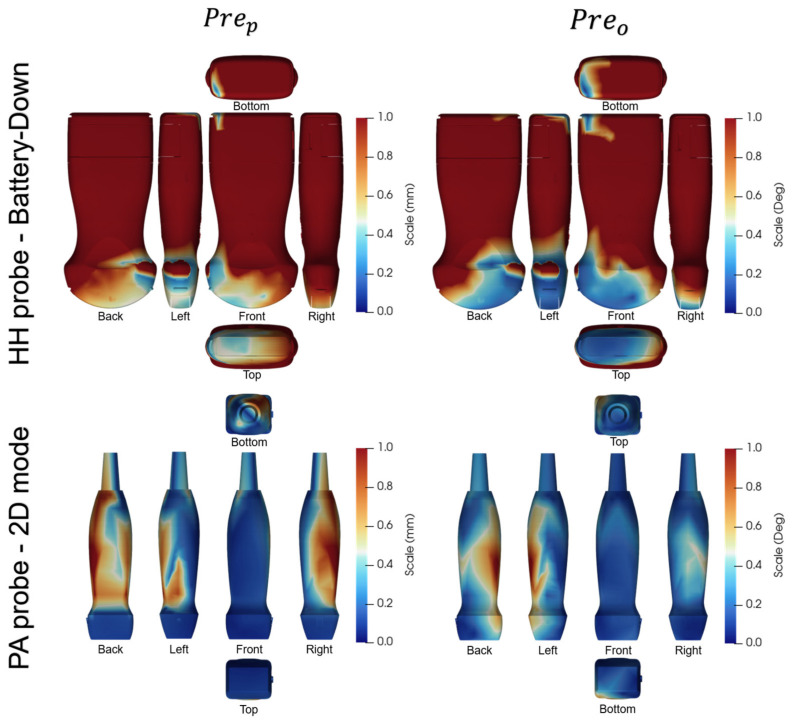
Spatial distribution of positional ($Pre_{p}$) and orientational ($Pre_{o}$) precision around US probes. Color maps illustrate the magnitude of positional ($Pre_{p}$, **left**) and orientational ($Pre_{o}$, **right**) precision across the surfaces of the HH probe (**top**) and the PA probe (**bottom**). Warm colors indicate higher variability (lower precision), while cool colors indicate more stable tracking regions with minimal EM interference. Multiple probe views are shown to highlight the spatial error distribution.

Figure 6 and Figure 7 present the positional and orientational precision of the EMTS under the different HH and PA probe configurations, respectively. These boxplots summarize the distribution of precision values across measurement locations and enable comparison between Empty, Off, and active configurations for each probe. Figure 8 illustrates the corresponding spatial distribution of precision across the probe surfaces during active operation, highlighting the regions of higher and lower tracking variability.

Regarding the PA probe, the median positional precision was 0.07 mm [0.06–0.08] and the orientational precision was 0.04° [0.02–0.05] in Empty. In the Off condition, precision remained within this low-magnitude baseline range (0.07 mm [0.06–0.09]; 0.04° [0.03–0.05]). When the probe was active, the median positional and orientational precision in 2D mode increased to 0.11 mm [0.08–0.56] and 0.06° [0.03–0.17], respectively. In 4D mode, they increased to 0.11 mm [0.08–0.22] and 0.06° [0.04–0.10]. Rotated configurations showed slightly higher medians but lower dispersion (2D-Rotated: 0.18 mm [0.09–0.39], 0.07° [0.04–0.16]; 4D-Rotated: 0.14 mm [0.10–0.20], 0.07° [0.05–0.09]). The Friedman test revealed significant global differences across the PA probe configurations (*p* < 0.001), and post hoc Wilcoxon signed-rank tests with Bonferroni correction confirmed higher variability in all active configurations compared with both Empty and Off conditions (Z = [−6.42 to −4.68], *p* < 0.001, r = [0.62–0.85]). Nevertheless, the absolute values remained submillimetric and subdegree across all active PA configurations. Significant differences were also found between 2D and 4D modes, and between 2D-Rotated and 4D-Rotated modes (Z = [3.18 to 3.63], *p* < 0.05; r = [0.42–0.48]). No significant differences were found between 2D and 2D-Rotated, or between 4D and 4D-Rotated (Z = [−0.27 to 0.37], *p* = 1.00; r < 0.05).

Regarding the HH probe, the median positional and orientational precision values were 0.07 mm [0.06–0.09] and 0.09° [0.04–0.18] in Empty, and remained within this baseline range in the Off condition (0.07 mm [0.06–0.10]; 0.04° [0.03–0.07]). In contrast, active conditions produced a substantial increase, with values of 2.45 mm [1.01–4.15] and 1.37° [0.39–2.74] in the Battery-Down configuration, and similar values in Battery-Up (2.46 mm [0.79–4.47] and 1.31° [0.39–3.09]). The Friedman test confirmed significant global differences across HH conditions (*p* < 0.001). Post hoc Wilcoxon signed-rank tests with Bonferroni correction showed that both active configurations exhibited significantly higher positional and orientational variability than both the Empty and the Off conditions (Z = [−12.80 to −12.26], *p* < 0.001, r = [0.83–0.87]), while no significant differences were detected between the two active orientations in positional and orientational precision (Z = [−1.39 to 0.64], *p* = 1.00, r = [0.04–0.09]).

The color maps in Figure 8 show that positional and orientational precision varied across the probe surface for both devices. The PA probe presented regions, particularly on the front surface, where a sensor could be positioned while maintaining the nominal precision of the system. In contrast, the HH probe presented extended regions of increased variability, indicating that stable tracking required positioning the EM sensor further from the probe surface.

### 3.2. Study 2—Effect of Probe–Sensor Separation for the Handheld US Probe

**Figure 9:**
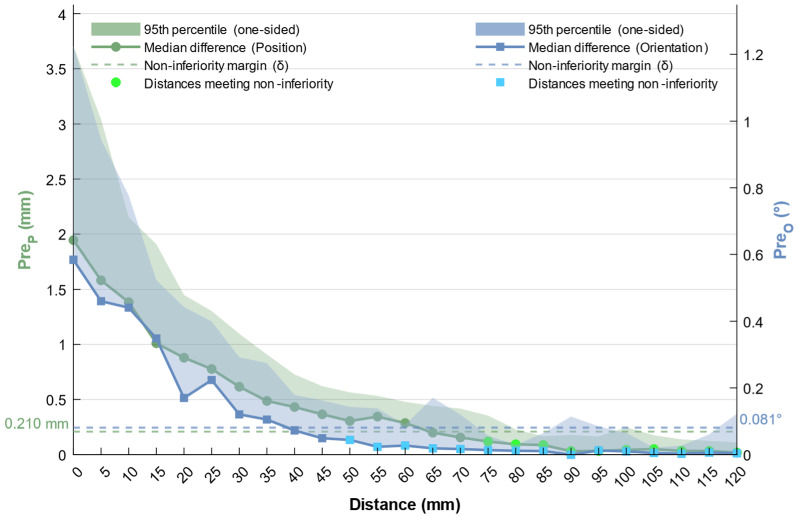
Non-inferiority analysis of positional ($Pre_{p}$) and orientational ($Pre_{o}$) precision. Median differences between US ON and Empty configurations are shown for positional precision (green, left axis) and orientational precision (blue, right axis), with the one-sided 95% confidence limits (shaded areas) and non-inferiority margins (δ = 0.21 mm; δ = 0.081°). Fluorescent markers denote distances where statistical analysis confirmed non-inferiority (*p* < 0.05).

Figure 9 illustrates the evolution of positional and orientational precision of the EM sensor as the distance from the US probe increases from 0 to 120 mm, in 5 mm increments. The lines show the median differences relative to the Empty configuration, together with one-sided 95th percentile confidence limits and the empirically derived non-inferiority margins (δ = 0.21 mm for position; δ = 0.081° for orientation). As expected, precision progressively improved with increasing distance from the US body, reaching a median positional difference of 0.12 mm [−0.01 to 0.35] at 75 mm and an orientational difference of 0.04° [−0.00 to 0.14] at 50 mm. The one-sided Wilcoxon signed-rank non-inferiority tests confirmed positional non-inferiority for distances ≥ 75 mm (*p* < 0.05) and orientational non-inferiority for distances ≥ 50 mm (*p* < 0.05), indicating that the effect of EM interference becomes negligible beyond these limits.

**Figure 10:**
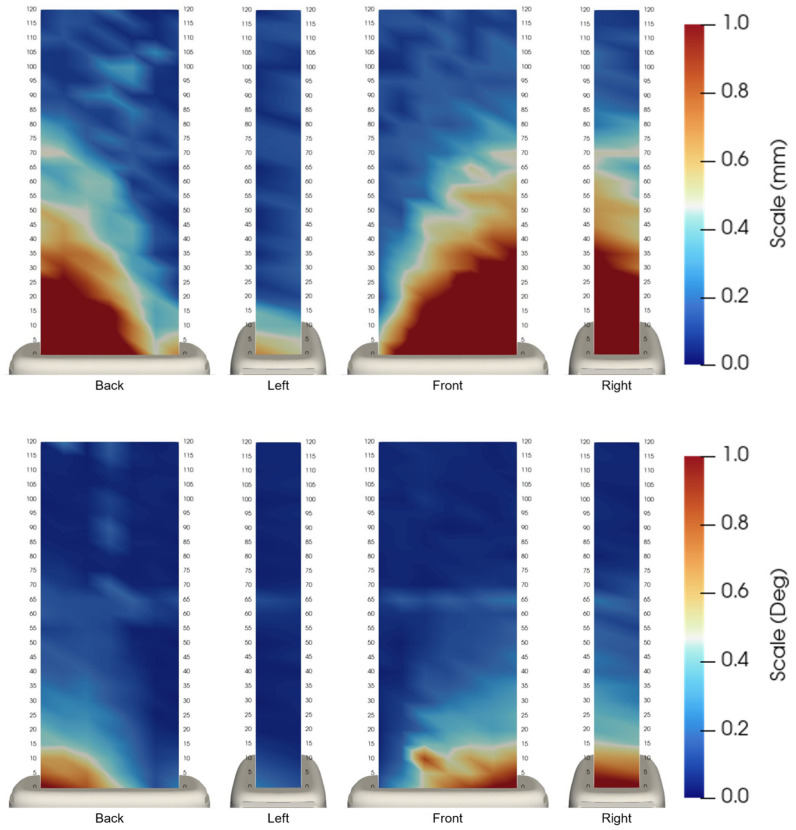
Spatial representation of positional ($Pre_{p}$) and orientational ($Pre_{o}$) precision with increasing distance from the active HH probe. Maps show positional precision (mm, (**top row**)) and orientational precision (°, (**bottom row**)); warm colors indicate higher variability, cool colours indicate more stable tracking regions. The continuous maps were interpolated from 525 regularly distributed measurement points (21 lateral positions × 25 distances). The probe is shown for spatial reference. The lateral axis indicates the distance from the HH probe surface in mm.

Figure 10 shows the spatial distribution of positional (top) and orientational (bottom) precision around the active HH probe. The color maps visually corroborate the statistical findings, illustrating a progressive reduction in tracking variability with increasing distance from the probe surface. Additionally, higher variability was observed on the right side of the probe, which is consistent with the asymmetric interference patterns identified in Study 1.

### 3.3. Study 3—Effect of US Probes During Simulated PCNL

**Figure 11:**
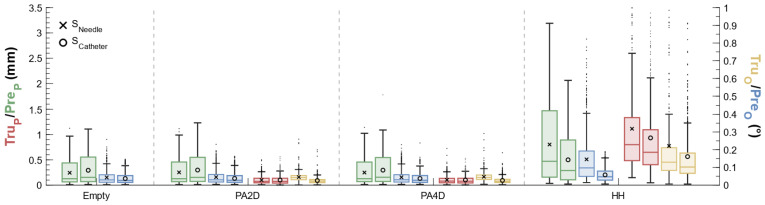
Positional and orientational precision ($Pre_{p},\;Pre_{o}$) and relative trueness (${Tru}_{p},\;{Tru}_{o}$) for both EM sensors ($S_{Needle}$ and $S_{Catheter}$) under Empty, PA2D, PA4D, and HH probe configurations. Boxes show the median and IQR, whiskers extend to the 5th–95th percentiles. Cross (x) and circle (o) markers indicate mean values for $S_{Needle}$ and $S_{Catheter}$, respectively.

Figure 11 summarizes the positional and orientational precision and trueness obtained with the two EM sensors ($S_{Needle}$ and $S_{Catheter}$) under different US configurations (Empty, PA2D, PA4D, and HH).

In the Empty configuration, median positional and orientational precisions were 0.15 mm [0.07–0.55] and 0.03° [0.02–0.05] for $S_{Catheter}$, and 0.13 mm [0.06–0.44] and 0.03° [0.02–0.06] for $S_{Needle}$. Under the PA probe configurations (PA2D and PA4D), both sensors maintained high precision, with median positional values below 0.16 mm [0.07–0.55] and orientational values of 0.03° [0.02–0.06]. In contrast, the HH probe configuration showed a noticeable decrease in precision, particularly in positional precision, and in both positional and orientational precision of the $S_{Needle}$. For the $S_{Catheter}$, median positional and orientational precision increased to 0.29 mm [0.11–0.89] and 0.05° [0.03–0.08], while $S_{Needle}$ exhibited 0.47 mm [0.16–1.47] and 0.10° [0.05–0.19], respectively. The Friedman test revealed a significant global effect between US configurations (*p* < 0.001). Post hoc Wilcoxon signed-rank tests with Bonferroni correction showed no significant differences between the Empty and PA probe configurations for either positional or orientational precision of $S_{Catheter}$ (Z = [−2.33 to −0.65], *p* = [0.20–1.00], r = [0.09–0.02]). For $S_{Needle}$, significant differences with small effect sizes were observed for positional and orientational precision (Z = [−6.41 to −3.78], *p* < 0.01, r = [0.15–0.25]). In contrast, the HH probe configuration exhibited large effect sizes in both $S_{Catheter}$ and $S_{Needle}$ positional and orientational precisions (Z = [−19.60 to −22.47], *p* < 0.01, r = [0.75–0.86]).

The trueness of position and orientation was assessed relative to the Empty configuration, as well as against the EMTS 5DOF manufacturer’s specification ($T_{spec}=0.7\;mm,0.2{}^{\circ}$). For the $S_{Catheter}$, median positional and orientational trueness values were 0.07 mm [0.04–0.14] and 0.02° [0.01–0.03] for PA2D, 0.08 mm [0.04–0.14] and 0.02° [0.01–0.03] for PA4D, and 0.65 mm [0.40–1.09] and 0.10° [0.07–0.18] for HH probe. For $S_{Needle}$, corresponding values were 0.07 mm [0.05–0.13] and 0.04° [0.03–0.05] for PA2D, 0.08 mm [0.05–0.13] and 0.04° [0.03–0.05] for PA4D, and 0.80 mm [0.48–1.34] and 0.13° [0.08–0.21] for HH probe. Non-inferiority Wilcoxon signed-rank tests (right-tailed) were applied to assess whether the tracking error exceeded the specification threshold. Both PA2D and PA4D configurations showed no significant deviation from the specification for both sensors (Z = [−22.50 to −22.51], *p* = 1.00, r = 0.87), confirming non-inferiority relative to the manufacturer-defined limits. For the HH probe, positional trueness exceeded the specification for the $S_{Catheter}$ and $S_{Needle}$ (Z = [1.67 to 8.28], *p* < 0.05, r = [0.03–0.06]), indicating a statistically significant increase in positional error, while orientation trueness remained within acceptable limits (Z = [−11.93 to −9.30], *p* = 1, r = [0.36–0.46]).

**Figure 12:**
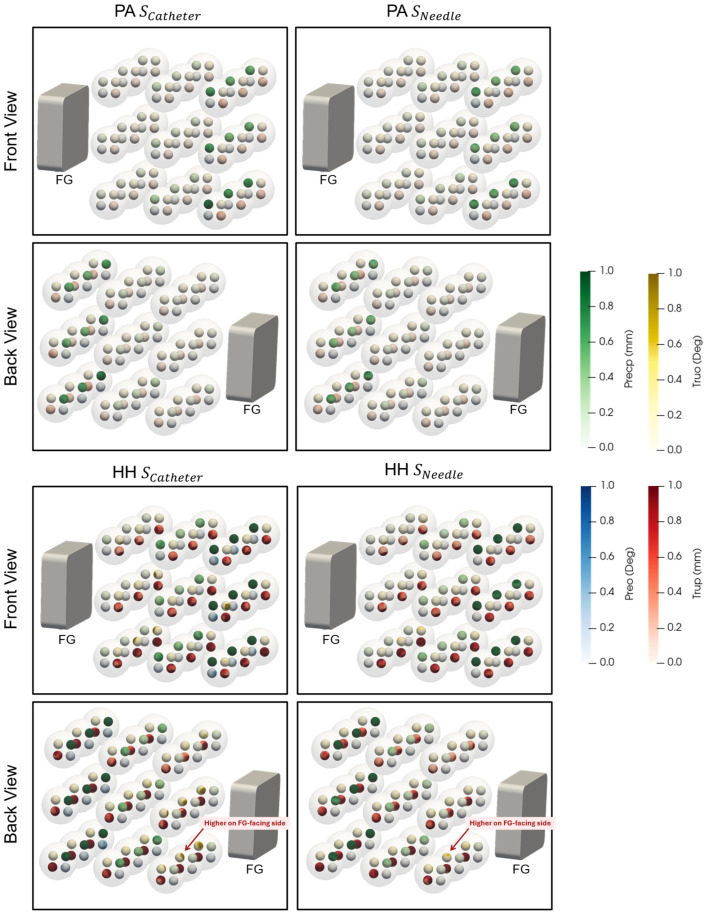
Spatial mapping of the positional and orientational precision ($Pre_{p},\;Pre_{o}$) and relative trueness (${Tru}_{p},\;{Tru}_{o}$) for both EM sensors ($S_{Catheter}$ and $S_{Needle}$) under PA2D and HH probe configurations. Each large translucent sphere represents one of the 27 support placements, containing four smaller spheres corresponding to the four metrics, spatially interpolated across the 25 measurement holes. The gray block represents the field generator (FG). Front and back views show the error distribution across the workspace, with annotations indicating higher errors on the FG-facing side under the HH configuration.

Figure 12 provides a spatial visualization of positional and orientational precision and trueness errors for both sensors ($S_{Catheter}$ and $S_{Needle}$) under the PA2D and HH probe configurations, for each sphere and each hole. Under PA2D, errors were homogeneously distributed across the workspace, with a slight expected increase in magnitude with distance from the FG. Under the HH probe, the same distance-dependent trend was observed. In addition, localized increases in error were concentrated on the FG-facing side of the measurement supports. Given the probe placement described in Section 2.3.5, these locations corresponded to configurations in which the HH probe was positioned between the FG and the tracked sensors.

### 3.4. EM-US Calibration

The calibration procedure estimated the temporal and spatial relationship between the EMTS and the US imaging plane for both US probes in 2D mode.

Phantom registration, performed once using the N-wire reference model, achieved a mean RMS fitting error of 0.83 mm, which served as the spatial reference for both probes. Temporal calibration resulted in synchronization of offsets of 69.63 ms for the PA probe and 83.85 ms for the HH probe.

For spatial calibration, the EM sensor was mounted on the frontal face of the PA probe, as determined in Study 1, and 75 mm from the top edge of the HH probe, according to Study 2 (Figure 5). Across ten repeated calibrations, the mean RMS calibration residuals were 0.92 ± 0.21 mm for the PA probe and 0.93 ± 0.19 mm for the HH probe.

## 4. Discussion

EMTS have become increasingly relevant in image-guided surgery because they provide continuous 3D localization without line-of-sight restrictions. However, in real operating-room environments, numerous devices may perturb the EM field, particularly when EMTS is combined with medical imaging modalities such as US. Several studies have investigated EM tracking accuracy under clinical conditions and in the presence of different surgical tools [7,16,17,18]. Collectively, these studies have established that US probes can affect EM tracking performance, but the present work extends this evidence through an integrated, device-specific evaluation combining dense spatial mapping along the probe bodies, quantitative assessment of probe–sensor distance, evaluation in a simulated PCNL configuration, and EM-US calibration using low-interference sensor-placement conditions.

The first experiment revealed that EM interference is highly probe-dependent and varies across the physical surface of each probe, confirming the importance of probe-specific characterization before clinical integration. The PA probe produced minimal distortions, maintaining submillimetric positional precision and subdegree orientational precision in all tested configurations. Although some increase in dispersion was observed compared with the Empty condition, indicating localized field distortions along the US probe body, these differences were small relative to the tracking variability observed for the HH probe. Both rotated configurations showed slightly higher medians but reduced dispersion, suggesting mild spatial asymmetry (Figure 8), consistent with the expectation that EM distortion increases when the probe lies between the FG and the tracked sensor [17]. However, no significant differences were observed between rotated and non-rotated configurations within the same imaging mode. Unlike mechanically actuated 3D/4D US probes previously reported to produce stronger interference due to internal motors [16,18], the evaluated PA probe is an active-matrix 3D/4D phased-array transducer without internal mechanical scanning components. This architecture is consistent with the absence of degradation when switching from 2D to 4D mode under the tested conditions. Although statistically significant differences were observed between modes, these reflected higher dispersion during 2D acquisition rather than deterioration during 4D. The responsible mode-dependent parameters were not isolated experimentally. Regardless of imaging mode, the spatial maps showed that regions around the acoustic window preserved nominal EM precision, supporting low-interference sensor placement directly on the probe without significant offset.

In contrast, the HH probe exhibited markedly higher EM interference, with median positional and orientational precision values exceeding 2.46 mm and 1.31°, respectively, in its active configurations, consistent with previous findings involving a wireless handheld US probe [21]. The Off condition helped distinguish passive probe presence from active probe operation. For both probes, the Off condition remained within the low-magnitude baseline range, whereas active operation was associated with significantly higher tracking variability. This indicates that the dominant disturbance, particularly for the HH probe, was associated with active operation rather than passive presence. A similar dependence on operating state was reported by Bonmati et al. [17], who observed greater positional and rotational jitter when an endoscopic US transducer was active than when it was switched off. From a hardware perspective, this pattern is consistent with architectural differences between the evaluated systems. The PA probe is connected to a console-based US system, in which most power supply, excitation, and processing functions are located outside the probe housing, whereas the HH probe integrates battery-powered electronics, power-management circuitry, imaging electronics, and wireless communication within the probe body. However, the present experiments do not isolate the relative contributions of individual subsystems, such as DC–DC power conversion, active imaging electronics, or wireless transmission. Therefore, the observed interference should be interpreted as associated with active HH probe operation rather than attributed to a specific internal source. Although Battery-Up and Battery-Down produced similar overall interference levels, slight asymmetry in the spatial error distribution was observed (Figure 8), indicating that interference was not uniformly distributed and that sensor placement should account for local distortion patterns. The spatial analysis showed higher distortion near the probe housing and lower distortion near the footprint, suggesting the footprint region as a candidate for sensor attachment when mechanically feasible. However, continuous probe manipulation during clinical use may intermittently position the probe between the FG and the sensor, increasing interference. These findings reinforce the importance of probe-specific characterization and clarify how changes in the relative probe–FG–sensor geometry during clinical use may lead to transient tracking instability.

Building upon these findings, the second experiment quantified how increasing the distance between the HH probe and an EM sensor mitigates field distortion. This configuration was chosen because mounting the sensor above the probe preserves probe-handling flexibility without obstructing the transducer footprint. The distance-response behavior confirmed the spatial trends observed earlier, including a mild but consistent right-sided asymmetry in the distortion field, consistent with the probe-specific design-related interference patterns observed in Study 1. As expected, the interference magnitude decreased progressively with increasing distance, and this trend is clearly illustrated in Figure 9. Statistical analysis demonstrated non-inferiority of positional and orientational precision relative to the Empty condition at distances of ≥75 mm and ≥50 mm, respectively. Pham et al. [21] similarly reported a distance-dependent influence of an active Clarius C3 HD3 scanner on EM tracking and selected a 150 mm probe–sensor separation to maintain tracking errors below 1 mm in their Polhemus G4 setup. Although maintaining a 75 mm separation is feasible in controlled environments, clinical integration requires careful consideration of probe length (~160 mm), typical PCNL imaging depths (80–120 mm [30,31]), and the FG volume (500×500×500 mm) to ensure full coverage within the FG workspace. Nevertheless, this configuration appears compatible with the spatial constraints of the tested PCNL-like setup, although ergonomic and workflow feasibility in clinical use remains to be evaluated. This underscores the need for awareness of probe positioning in hybrid EM-US systems and suggests that automated or passive compensation strategies, such as the witness-sensor approaches described by Sadjadi et al. [19] or distortion-modeling strategies proposed by Cavaliere et al. [20], could further improve robustness when handheld probes are used. Overall, this experiment supports the asymmetric distortion pattern identified earlier and defines quantitative distance thresholds for preserving tracking stability, providing device-specific information for integrating the evaluated handheld probe into EM-tracked surgical workflows.

A practical consequence of the 75 mm separation identified for the HH probe is the need to position the EM sensor at an offset from the probe body, which introduces a trade-off between EM tracking performance and clinical ergonomics. A rigid extension of this length could increase the effective lever arm between the operator’s grip and the sensor, potentially altering probe hand-feel and balance, increasing torque during manipulation, and inducing cantilever vibration or deflection during rapid scanning motions. These mechanical effects could affect both operator comfort and the stability of the tracked image plane, since orientation uncertainty is amplified through the lever-arm effect, which may in turn increase image localization error, particularly during probe rotation. Sensor placement must therefore balance the reduction in local EM interference against ergonomic and mechanical constraints. Importantly, these effects were not quantified in the present controlled setup and remain to be evaluated under realistic, dynamic clinical handling. Optimizing the mechanical design of the sensor mount, including its length, rigidity, and mass distribution, is therefore an important direction for future work.

The third study evaluated how each probe affects EMTS during a simulated PCNL procedure. Consistent with earlier findings, the PA probe maintained positional and orientational precision statistically indistinguishable from the Empty condition across both 2D and 4D imaging modes, reinforcing the interpretation that phased-array probes without mechanical actuation may generate lower interference than mechanically actuated probes [16,17]. In contrast, the HH probe produced a clear degradation in both positional and orientational precision, particularly when placed between the EM sensors and the FG, a behavior consistent with the attenuation effects described by Fonseca et al. [18] for mechanically actuated probes, and known characteristics of the EM field, including increased distortion near conductive housings and reduced precision at the boundaries of the tracking volume [22,27,32]. Despite statistical differences relative to the Empty condition, median tracking errors remained within commonly cited tolerance ranges for renal puncture from a tracking-performance perspective, given typical calyx dimensions and procedural tolerances [3,4,33]. Overall, these results indicate that the PA probe produced minimal tracking disturbance during the simulated PCNL workflow, while the HH probe introduced stronger, spatially localized distortions consistent with the probe-specific interference patterns identified in Study 1. Importantly, accurate tracking can still be achieved, provided that operators avoid known high-interference regions and avoid configurations in which the probe is positioned between the FG and the tracked sensors.

The final experiment evaluated whether EM-US calibration could be performed using the sensor-placement strategies identified in the previous studies. Both probes produced submillimetric RMS calibration residuals when sensors were mounted in low-interference locations, in line with the values reported for state-of-the-art N-wire calibration pipelines [34,35,36], underscoring the importance of optimal sensor placement during the calibration process. Although the calibration was conducted under ideal static conditions, including a stable environment, rigid phantom, and controlled probe motion, the comparable residuals obtained for both probes support the use of identified low-interference sensor locations for EM-US calibration. In particular, the evaluated HH probe produced RMS calibration residuals comparable to those of the conventional PA probe when the sensor was mounted in a low-interference region. However, these residuals reflect the internal fitting consistency of the calibration procedure and should not be interpreted as an independent measure of Target Registration Error (TRE) or clinical navigation accuracy.

Although the experiments provide a characterization of EM-US interactions, several limitations must be acknowledged. First, all studies were conducted under controlled laboratory conditions and did not include metallic instruments, patient anatomy, or other sources of interference commonly present in the operating room. Therefore, the experimental setup should be interpreted as a controlled technical assessment of tracking performance rather than as a complete representation of clinical use. From a practical workflow perspective, the size of the HH probe, the added sensor housing, the need for sensor offset, and the requirement to maintain the probe, sensors, and target region within the EM tracking volume may impose ergonomic and workflow constraints that were not evaluated in this study. A related consideration is that attaching conventional wired EM sensors partially reduces the cable-free advantage of handheld wireless US probes. However, the EM sensor cable is substantially thinner and less restrictive than a conventional US probe cable, and the handheld platform still avoids the need for a large US console in the operating room. Thus, although this is not a fully wireless navigation solution, it may still reduce workspace constraints compared with conventional cart-based US systems. Future integrated or wireless tracking sensors should be evaluated separately, as they may introduce additional interference sources. Second, all measurements were acquired under static probe and sensor configurations. Although Study 3 reproduced a PCNL-like spatial arrangement, it did not reproduce the continuous probe and instrument manipulation occurring during clinical scanning and puncture. Although the two tested probe orientations did not differ significantly in the global precision metrics, the spatial maps showed mild local asymmetry in the interference distribution. Therefore, continuous probe manipulation, tilting, and repositioning could dynamically move the tracked sensors through localized regions of higher and lower distortion by changing the relative geometry between the probe, FG, and sensors. The HH probe could also be transiently positioned between the FG and the sensors, potentially producing time-varying tracking instability that was not captured by the static measurements. Dynamic, motion-resolved characterization under clinically realistic PCNL conditions is therefore required to quantify these transient effects. Third, inter-operator variability was not assessed, and differences in probe handling, grip, or rotation patterns could influence interference distribution. Fourth, the results are specific to the GE 4VC-D, Clarius C3 HD, and Aurora V2 systems; other probe designs or field generators may exhibit different interaction profiles. Finally, although the experimental protocol included an Off condition to distinguish passive probe presence from active operation, electronic factors specific to the HH probe, such as battery level, power-management operation, imaging electronics, and wireless transmission state, were not isolated or systematically varied, although they may influence interference magnitude. These considerations suggest that while the present findings provide device-specific baseline guidance, further validation in realistic, dynamic clinical environments remains necessary.

Overall, this work provides an experimental framework for understanding and mitigating EM interference in hybrid EM-US systems. The results demonstrate that EM distortion is both architecture-dependent and spatially localized, but can be mitigated through informed sensor placement, adequate probe orientation, and appropriate distance management. The PA probe produced minimal tracking disturbances under the tested conditions, whereas the HH probe introduced larger but spatially localized distortions that could be reduced through sensor placement, probe orientation, and distance management. These insights provide technical evidence for clinicians and engineers integrating EM-US systems in percutaneous procedures such as PCNL, and motivate further evaluation of ergonomic, workflow, and environmental constraints in dynamic, real operating-room conditions.

## 5. Conclusions

This work extends previous investigations of US-induced EM interference through an integrated, device-specific characterization combining spatial mapping along the probe bodies, quantitative probe–sensor distance assessment, evaluation in a simulated PCNL configuration, and EM-US calibration under low-interference sensor-placement conditions. Across all experiments, the PA probe produced minimal tracking disturbance under the tested configurations, while the HH probe exhibited localized, architecture-dependent distortions that could be reduced through informed sensor placement, appropriate probe–FG–sensor geometry, and adherence to minimum distance thresholds.

Both probes produced submillimetric RMS calibration residuals under controlled conditions when sensors were positioned in low-interference regions, supporting the use of the identified sensor-placement strategy for hybrid EM-US calibration, including with the evaluated HH probe. These findings provide experimental evidence for designing EM-compatible US setups and support future development of EM-based navigation in PCNL and related minimally invasive procedures. Future work should validate these findings in dynamic, clinically realistic environments where motion, anatomical variability, and surrounding equipment may introduce additional EM field perturbations.

As a final remark, to facilitate the reproduction of this work, the Supplementary Materials provides detailed information on the technical setup, CAD files for the experimental fixtures, spatial-map results in VTK format, and complementary tracking-variability metrics. These resources support reproducibility and enable further analysis of the probe-dependent interference patterns reported in this study.

## Data Availability

The data supporting the findings of this study are provided as Supplementary Materials.

## Supplementary Materials

The following supporting information can be downloaded at: https://www.mdpi.com/article/10.3390/s26134096/s1. Supplementary Material S1 provides technical setup guidelines for EM-US integration in PCNL and related interventions. Supplementary Material S2 contains all CAD files required to produce fixtures, capsules, and positioning jigs. Supplementary Material S3 provides the spatial map results as VTK files. Supplementary Material S4 provides complementary tracking-variability metrics, including RMS, P95, and maximum positional and orientational deviations.

## Abbreviations

The following abbreviations are used in this manuscript:

2D	Two-dimensional
3D	Three-dimensional
4D	Four-dimensional
API	Application programming interface
CAD	Computer-aided design
DOF	Degrees of freedom
EM	Electromagnetic
EMTS	Electromagnetic tracking system
FDM	Fused deposition modeling
FG	Field generator
FOV	Field of view
fps	Frames per second
HH	Evaluated Clarius C3 HD Scanner
IQR	Interquartile range
NDI	Northern Digital Inc.
PA	Evaluated GE 4VC-D
PA2D	PA probe operating in 2D mode
PA4D	PA probe operating in 4D mode
PCNL	Percutaneous nephrolithotomy
PLA	Polylactic acid
RMS	Root mean square
TRE	Target registration error
US	Ultrasound
VTK	Visualization Toolkit
