# Supplementary material for: Characterization of Ultrasound Probe-Dependent Interference in Electromagnetic Tracking for Image-Guided Procedures
Source: Sensors (Basel). 2026 Jun 27;26(13):4096. doi: 10.3390/s26134096 (PMC13363777; doi:10.3390/s26134096)
Supplement: Supplementary file 1 [file sensors-26-04096-s001.zip › Supplementary Material S1/S1 - Practical Setup Guidelines For EM-US integration in PCNL.pdf]

Supplementary Material S1

Practical Setup Guidelines for EM–US Integration in PCNL and Image-Guided Interventions

This supplementary document provides practical recommendations for clinicians, biomedical engineers, and operating-room staff aiming to correctly configure electromagnetic (EM) tracking and ultrasound (US) devices during percutaneous nephrolithotomy (PCNL) and other image-guided procedures. All guidelines are derived from the experimental results reported in this study. An example of a recommended EM–US configuration for PCNL is shown in Figure S1, which provides visual context for the setup principles described below.

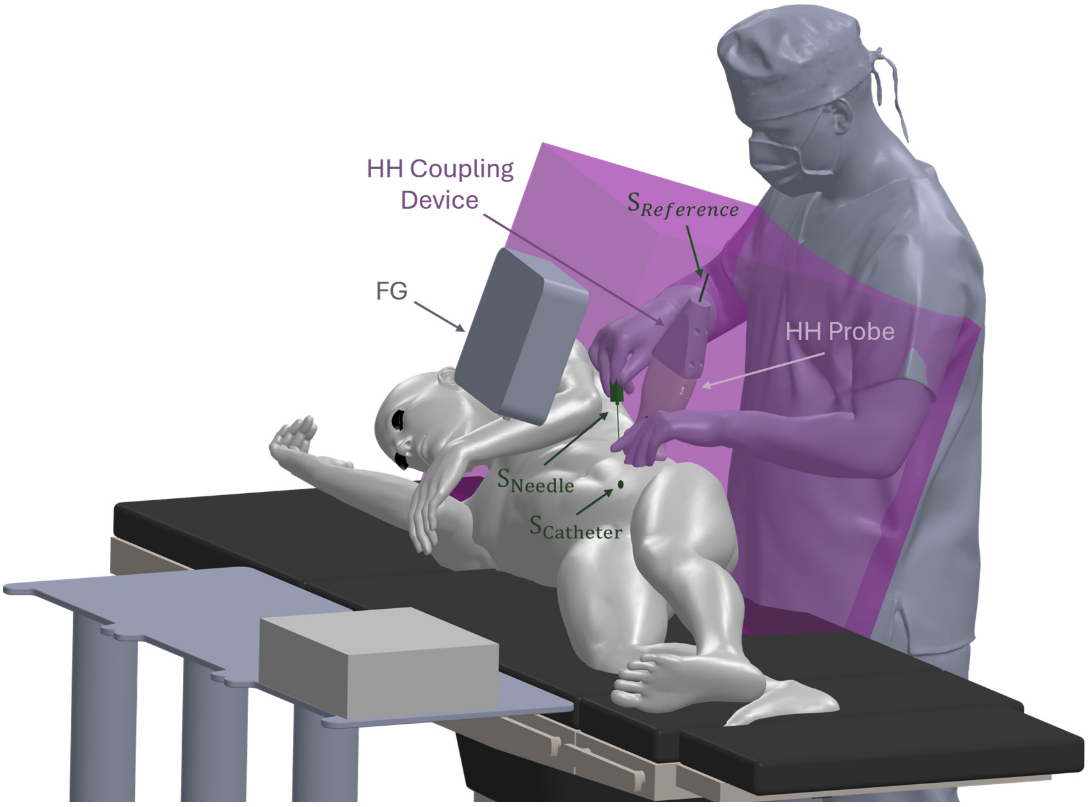

Figure S1 - Example PCNL-like configuration showing recommended positioning of the field generator (FG), the handheld ultrasound probe (HH probe), the coupling device, and the electromagnetic (EM) sensors (S_{Needle}, $S_{Catheter}$, $S_{Reference}$) within the effective EM tracking volume. This setup illustrates the key spatial relationships required to minimize EM interference during hybrid EM-US guidance.

1. <u>Field Generator (FG) / EM Tracking System (EMTS)</u>
   - Position the FG so that the probe and target anatomy remain near the central region of the EMTS tracking volume.
   - Maintain a direct, unobstructed path between the FG and all EM sensors.
   - Avoid placing the US probe or instrument between the FG and the sensors.
   - Maximize distance between the FG and metallic tables, trays, cables, C-arm components, monitors, power supplies or external batteries.
   - Secure the FG to prevent vibration or unintended movement.

2. <u>Ultrasound Probes</u>

- GE 4VC-D (Phased Array (PA) probe).
  - Fully compatible with EMTS under 2D and 4D imaging.
  - No minimum safe-probe distance is required.
  - Avoid only interposing the probe directly between FG and sensor.
- Clarius C3HD (Handheld (HH) probe).
  - Due to the internal battery/electronics/wireless modules, the probe exhibits localized EM interference.
  - Safe-distance guidance:
    - Positional precision recovery: ≥ 75 mm separation from the battery region;
    - Orientational precision recovery: ≥ 50 mm;
    - Greater distances may reduce ergonomics and should be balanced against handling comfort;
    - Shorter distances can still be used, but expect increased jitter and small tracking offsets.
  - Recommended mounting:
    - Attach the EM sensor ensuring ≥ 75 mm from the upper housing.
    - Avoid probe rotations that place battery housing between the FG and the sensor;
    - If using an adhesive mount, orient the sensor towards the footprint side, where interference is lowest.

3. <u>PCNL Workflow Considerations</u>
   - Ensure EM sensors (needle and catheter) remain well within the FG tracking volume.
   - With the PA probe:
     - No manipulation restrictions.
     - Probe can be rotated freely without measurable effect on EM stability.
   - With the HH probe:
     - Maintain the probe outside mapped high-interference zones.
     - Tilt or orient the probe so that the battery compartment is away from the FG.
     - If tracking jitter increases, verify:
       - Probe alignment relative to FG;
       - Distance between sensor and probe;
       - Presence of external metallic objects.

These guidelines promote reproducible, accurate EM-US integration, support safer renal access during PCNL, and facilitate translational adoption of hybrid navigation systems.
