# Supplementary material for: Characterization of Ultrasound Probe-Dependent Interference in Electromagnetic Tracking for Image-Guided Procedures
Source: Sensors (Basel). 2026 Jun 27;26(13):4096. doi: 10.3390/s26134096 (PMC13363777; doi:10.3390/s26134096)
Supplement: Supplementary file 1 [file sensors-26-04096-s001.zip › Supplementary Material S4/S4-Complementary_Tracking_Variability_Metrics.pdf]

Supplementary Materials

Characterization of Ultrasound Probe-Dependent Interference in Electromagnetic Tracking for Image-Guided Procedures

Complementary tracking-variability metrics

The supplementary tables report complementary positional and orientational tracking-variability metrics for Studies 1–3. At each measurement location, positional and orientational precision (root-mean-square, RMS) was computed as defined in the main manuscript. The 95th percentile (P95) and maximum (worst-case) sample-wise deviations from the corresponding mean position or orientation were additionally calculated as complementary descriptors of the same per-location distributions. P95 represents the value below which 95% of the sample-wise deviations fall, whereas the maximum represents the largest observed deviation. Unless otherwise stated, all three metrics are summarized across valid measurement locations as median [Q1–Q3].

Table S1. Complementary positional and orientational tacking-variability metrics for Study 1 (spatial characterization of US probe-induced interference). Values are summarized across measurement locations as median [Q1-Q3].

Probe	Configuration	n	Positional (mm)			Orientational (°)		
			RMS	P95	Maximum	RMS	P95	Maximum
**HH**	Empty	218	0.07 [0.06-0.09]	0.11 [0.09-0.14]	0.18 [0.14-0.26]	0.09 [0.04-0.18]	0.17 [0.07-0.32]	0.21 [0.10-0.38]
**HH**	Off	218	0.07 [0.06-0.10]	0.12 [0.09-0.16]	0.19 [0.13-0.28]	0.04 [0.03-0.07]	0.08 [0.04-0.13]	0.11 [0.07-0.19]
**HH**	Battery-Down	218	2.45 [1.01-4.15]	4.45 [1.72-7.58]	5.75 [2.26-9.41]	1.38 [0.39-2.74]	2.68 [0.76-5.02]	3.28 [1.04-6.29]
**HH**	Battery-Up	218	2.46 [0.78-4.47]	4.19 [1.36-8.21]	5.26 [1.68-10.29]	1.31 [0.39-3.09]	2.37 [0.74-6.02]	2.94 [1.03-7.51]
**PA**	Empty	57	0.07 [0.06-0.08]	0.11 [0.09-0.14]	0.18 [0.12-0.26]	0.04 [0.02-0.05]	0.06 [0.04-0.09]	0.09 [0.06-0.13]
**PA**	Off	57	0.07 [0.06-0.09]	0.11 [0.09-0.14]	0.17 [0.14-0.21]	0.04 [0.03-0.05]	0.07 [0.04-0.10]	0.08 [0.06-0.14]
**PA**	2D	57	0.11 [0.08-0.56]	0.17 [0.13-0.89]	0.33 [0.18-1.22]	0.06 [0.03-0.18]	0.11 [0.06-0.34]	0.18 [0.09-0.51]
**PA**	2D-Rotated	57	0.18 [0.09-0.39]	0.25 [0.14-0.76]	0.41 [0.19-1.16]	0.07 [0.04-0.16]	0.12 [0.07-0.26]	0.16 [0.10-0.44]
**PA**	4D	57	0.11 [0.09-0.22]	0.17 [0.13-0.34]	0.30 [0.19-0.60]	0.06 [0.04-0.10]	0.10 [0.06-0.17]	0.14 [0.09-0.23]
**PA**	4D-Rotated	57	0.14 [0.10-0.20]	0.21 [0.15-0.31]	0.32 [0.21-0.74]	0.07 [0.05-0.09]	0.11 [0.07-0.15]	0.16 [0.11-0.26]

Note. *RMS, P95, and maximum deviations were first calculated from the repeated samples acquired at each measurement location and then summarized across locations as median [Q1-Q3]. n denotes the number of measurement locations per configuration.*

Table S2. Complementary positional and orientational tacking-variability metrics for Study 2 (distance-dependent characterization). For each distance, metrics were computed at each measurement position and summarized across valid positions as median [Q1-Q3].

Distance (mm)	Condition	Positional (mm)			Orientational (°)		
		RMS	P95	Maximum	RMS	P95	Maximum
0	Empty	0.09 [0.03-0.10]	0.05 [0.04-0.06]	0.57 [0.10-0.63]	0.03 [0.03-0.03]	0.05 [0.04-0.06]	0.08 [0.07-0.12]
	US-On	2.04 [1.04-3.47]	4.43 [1.90-6.15]	4.66 [2.67-8.20]	0.61 [0.31-0.98]	1.15 [0.56-1.93]	1.40 [0.74-2.65]
5	Empty	0.09 [0.04-0.09]	0.06 [0.05-0.07]	0.58 [0.13-0.60]	0.04 [0.03-0.04]	0.06 [0.05-0.07]	0.08 [0.07-0.15]
	US-On	1.68 [0.73-2.34]	3.15 [1.45-4.36]	3.73 [1.93-5.93]	0.51 [0.21-0.75]	0.95 [0.40-1.33]	1.13 [0.45-1.88]
10	Empty	0.09 [0.04-0.10]	0.06 [0.05-0.06]	0.59 [0.12-0.63]	0.03 [0.03-0.04]	0.06 [0.05-0.07]	0.08 [0.06-0.11]
	US-On	1.47 [0.67-1.97]	2.57 [1.30-3.63]	3.17 [2.00-5.18]	0.48 [0.17-0.64]	0.75 [0.31-1.25]	1.18 [0.40-1.55]
15	Empty	0.09 [0.09-0.10]	0.05 [0.05-0.07]	0.62 [0.58-0.68]	0.03 [0.03-0.04]	0.06 [0.05-0.07]	0.10 [0.07-0.13]
	US-On	1.11 [0.53-1.58]	2.29 [0.98-2.87]	2.79 [1.11-3.70]	0.38 [0.15-0.44]	0.66 [0.31-0.81]	0.83 [0.37-1.12]
20	Empty	0.09 [0.03-0.10]	0.05 [0.05-0.06]	0.63 [0.10-0.70]	0.03 [0.03-0.04]	0.06 [0.05-0.07]	0.08 [0.07-0.10]
	US-On	0.97 [0.43-1.30]	1.60 [0.84-2.34]	2.13 [1.05-3.08]	0.25 [0.11-0.35]	0.46 [0.24-0.68]	0.62 [0.26-0.89]
25	Empty	0.09 [0.03-0.10]	0.05 [0.05-0.07]	0.61 [0.08-0.66]	0.04 [0.03-0.06]	0.07 [0.05-0.11]	0.15 [0.08-0.20]
	US-On	0.88 [0.44-1.14]	1.63 [0.79-2.08]	2.10 [1.07-2.55]	0.27 [0.10-0.31]	0.45 [0.20-0.55]	0.59 [0.26-0.89]
30	Empty	0.10 [0.03-0.11]	0.06 [0.05-0.07]	0.67 [0.11-0.72]	0.04 [0.03-0.09]	0.07 [0.06-0.16]	0.12 [0.09-0.22]
	US-On	0.67 [0.37-0.90]	1.17 [0.67-1.49]	1.41 [0.88-2.14]	0.18 [0.09-0.26]	0.34 [0.16-0.51]	0.42 [0.22-0.69]
35	Empty	0.09 [0.03-0.10]	0.05 [0.05-0.07]	0.64 [0.08-0.70]	0.03 [0.03-0.05]	0.06 [0.05-0.09]	0.09 [0.08-0.14]
	US-On	0.58 [0.29-0.78]	1.13 [0.59-1.48]	1.42 [0.81-2.29]	0.16 [0.06-0.22]	0.31 [0.13-0.45]	0.39 [0.19-0.52]
40	Empty	0.10 [0.03-0.11]	0.06 [0.05-0.06]	0.66 [0.07-0.74]	0.04 [0.04-0.05]	0.07 [0.06-0.10]	0.11 [0.08-0.13]
	US-On	0.52 [0.24-0.63]	0.87 [0.34-1.20]	1.31 [0.94-1.52]	0.13 [0.05-0.19]	0.26 [0.09-0.36]	0.30 [0.15-0.46]
45	Empty	0.11 [0.08-0.11]	0.06 [0.05-0.07]	0.71 [0.48-0.74]	0.04 [0.03-0.05]	0.06 [0.06-0.09]	0.10 [0.07-0.16]
	US-On	0.47 [0.17-0.61]	0.78 [0.27-1.09]	0.98 [0.72-1.49]	0.12 [0.06-0.16]	0.20 [0.11-0.29]	0.25 [0.16-0.42]
50	Empty	0.10 [0.04-0.12]	0.06 [0.05-0.06]	0.69 [0.11-0.77]	0.04 [0.03-0.05]	0.07 [0.06-0.08]	0.12 [0.08-0.17]
	US-On	0.42 [0.16-0.51]	0.71 [0.24-0.87]	0.92 [0.73-1.29]	0.12 [0.05-0.15]	0.21 [0.09-0.27]	0.28 [0.15-0.38]
55	Empty	0.10 [0.03-0.13]	0.06 [0.05-0.07]	0.65 [0.07-0.82]	0.04 [0.03-0.05]	0.07 [0.05-0.09]	0.10 [0.08-0.14]
	US-On	0.41 [0.17-0.49]	0.72 [0.23-0.82]	0.89 [0.78-1.08]	0.11 [0.06-0.13]	0.19 [0.10-0.25]	0.26 [0.16-0.32]
60	Empty	0.11 [0.04-0.13]	0.08 [0.06-0.10]	0.68 [0.13-0.77]	0.08 [0.07-0.11]	0.15 [0.11-0.19]	0.19 [0.12-0.26]
	US-On	0.41 [0.14-0.44]	0.64 [0.21-0.82]	0.90 [0.71-1.06]	0.13 [0.09-0.14]	0.21 [0.15-0.26]	0.24 [0.18-0.29]
65	Empty	0.10 [0.04-0.12]	0.07 [0.06-0.08]	0.68 [0.08-0.78]	0.12 [0.10-0.19]	0.21 [0.16-0.33]	0.25 [0.21-0.41]
	US-On	0.26 [0.14-0.41]	0.58 [0.20-0.72]	0.86 [0.76-0.91]	0.14 [0.13-0.18]	0.28 [0.21-0.31]	0.32 [0.25-0.40]

Distance (mm)	Condition	Positional (mm)			Orientational (°)		
		RMS	P95	Maximum	RMS	P95	Maximum
70	Empty	0.11 [0.04-0.12]	0.07 [0.06-0.09]	0.75 [0.10-0.83]	0.04 [0.04-0.05]	0.07 [0.06-0.08]	0.11 [0.09-0.14]
	US-On	0.20 [0.16-0.47]	0.32 [0.20-0.71]	0.82 [0.75-0.89]	0.06 [0.05-0.09]	0.10 [0.08-0.21]	0.15 [0.12-0.31]
75	Empty	0.11 [0.04-0.12]	0.07 [0.06-0.07]	0.74 [0.13-0.81]	0.04 [0.03-0.05]	0.07 [0.06-0.08]	0.10 [0.08-0.13]
	US-On	0.20 [0.15-0.26]	0.30 [0.21-0.60]	0.81 [0.75-0.91]	0.06 [0.05-0.07]	0.09 [0.08-0.13]	0.20 [0.11-0.25]
80	Empty	0.11 [0.05-0.13]	0.08 [0.07-0.09]	0.76 [0.17-0.83]	0.04 [0.04-0.05]	0.07 [0.06-0.09]	0.11 [0.10-0.14]
	US-On	0.17 [0.15-0.23]	0.25 [0.19-0.37]	0.83 [0.60-0.95]	0.06 [0.05-0.06]	0.10 [0.09-0.11]	0.15 [0.11-0.20]
85	Empty	0.06 [0.04-0.13]	0.07 [0.06-0.08]	0.20 [0.10-0.87]	0.05 [0.04-0.05]	0.08 [0.08-0.09]	0.11 [0.09-0.17]
	US-On	0.17 [0.13-0.18]	0.21 [0.18-0.24]	0.84 [0.31-0.91]	0.06 [0.05-0.06]	0.09 [0.09-0.11]	0.13 [0.12-0.20]
90	Empty	0.13 [0.05-0.13]	0.08 [0.07-0.09]	0.79 [0.14-0.87]	0.05 [0.04-0.06]	0.08 [0.07-0.10]	0.11 [0.09-0.21]
	US-On	0.15 [0.12-0.19]	0.20 [0.17-0.26]	0.79 [0.25-0.90]	0.05 [0.04-0.06]	0.08 [0.07-0.10]	0.13 [0.10-0.20]
95	Empty	0.08 [0.04-0.13]	0.08 [0.07-0.08]	0.29 [0.09-0.87]	0.05 [0.04-0.06]	0.08 [0.08-0.10]	0.14 [0.10-0.17]
	US-On	0.15 [0.14-0.16]	0.18 [0.15-0.26]	0.77 [0.25-0.85]	0.06 [0.05-0.06]	0.09 [0.08-0.11]	0.14 [0.12-0.18]
100	Empty	0.05 [0.04-0.13]	0.07 [0.06-0.09]	0.15 [0.10-0.86]	0.05 [0.05-0.06]	0.09 [0.08-0.11]	0.13 [0.10-0.16]
	US-On	0.14 [0.09-0.18]	0.17 [0.14-0.21]	0.73 [0.17-0.89]	0.05 [0.05-0.07]	0.09 [0.08-0.12]	0.16 [0.11-0.23]
105	Empty	0.11 [0.04-0.14]	0.08 [0.06-0.09]	0.75 [0.11-0.89]	0.05 [0.04-0.05]	0.09 [0.07-0.10]	0.12 [0.10-0.15]
	US-On	0.14 [0.09-0.17]	0.15 [0.13-0.17]	0.76 [0.28-0.93]	0.05 [0.05-0.06]	0.09 [0.08-0.10]	0.12 [0.10-0.15]
110	Empty	0.11 [0.04-0.14]	0.06 [0.06-0.08]	0.77 [0.08-0.93]	0.05 [0.04-0.06]	0.09 [0.08-0.10]	0.11 [0.09-0.18]
	US-On	0.13 [0.08-0.15]	0.15 [0.12-0.17]	0.38 [0.16-0.90]	0.05 [0.05-0.06]	0.09 [0.08-0.11]	0.12 [0.10-0.15]
115	Empty	0.05 [0.04-0.13]	0.07 [0.06-0.08]	0.14 [0.07-0.84]	0.05 [0.04-0.06]	0.09 [0.08-0.09]	0.11 [0.09-0.13]
	US-On	0.14 [0.10-0.15]	0.14 [0.11-0.16]	0.82 [0.18-0.96]	0.06 [0.05-0.06]	0.10 [0.08-0.11]	0.13 [0.10-0.16]
120	Empty	0.12 [0.04-0.14]	0.07 [0.06-0.08]	0.80 [0.07-0.93]	0.05 [0.05-0.06]	0.09 [0.08-0.11]	0.13 [0.10-0.19]
	US-On	0.11 [0.08-0.15]	0.13 [0.11-0.16]	0.24 [0.15-0.91]	0.06 [0.05-0.07]	0.10 [0.08-0.11]	0.13 [0.11-0.15]

Note. *Metrics were calculated independently at each measurement position and summarized across valid positions at each distance as median [Q1-Q3]. These complementary metrics do not replace the RMS-based non-inferiority analysisor alter the distance thresholds reported in the main manuscript.*

Table S3. Complementary positional and orientational tacking-variability metrics for Study 3 (simulated PCNL configuration). Values are summarized across valid measurement locations as median [Q1-Q3].

Configuration	Condition	Nominal n	Positional (mm)			Orientational (°)		
			RMS	P95	Maximum	RMS	P95	Maximum
Clarius - Needle	Empty	675	0.13 [0.07-0.42]	0.21 [0.12-0.68]	0.34 [0.18-0.97]	0.03 [0.02-0.05]	0.05 [0.03-0.09]	0.08 [0.04-0.15]
Clarius - Needle	HH	675	0.47 [0.16-1.47]	0.79 [0.28-2.42]	1.21 [0.39-3.51]	0.10 [0.05-0.20]	0.17 [0.09-0.34]	0.24 [0.11-0.51]
Clarius - Catheter	Empty	675	0.13 [0.07-0.44]	0.22 [0.11-0.73]	0.33 [0.16-1.02]	0.02 [0.02-0.04]	0.04 [0.03-0.07]	0.05 [0.03-0.10]
Clarius - Catheter	HH	675	0.29 [0.11-0.89]	0.48 [0.19-1.47]	0.70 [0.26-2.08]	0.05 [0.03-0.08]	0.08 [0.05-0.14]	0.12 [0.07-0.20]
GE - Needle	Empty	675	0.13 [0.06-0.44]	0.21 [0.11-0.72]	0.32 [0.16-1.00]	0.03 [0.02-0.06]	0.05 [0.03-0.10]	0.08 [0.04-0.15]
GE - Needle	PA2D	675	0.13 [0.07-0.46]	0.21 [0.11-0.74]	0.32 [0.17-1.02]	0.03 [0.02-0.06]	0.05 [0.03-0.11]	0.08 [0.04-0.15]
GE - Needle	PA4D	675	0.13 [0.07-0.46]	0.21 [0.11-0.75]	0.33 [0.16-1.02]	0.03 [0.02-0.06]	0.05 [0.03-0.10]	0.08 [0.04-0.15]
GE - Catheter	Empty	675	0.15 [0.07-0.55]	0.25 [0.11-0.92]	0.35 [0.17-1.17]	0.03 [0.02-0.05]	0.05 [0.03-0.10]	0.07 [0.04-0.13]
GE - Catheter	PA2D	675	0.15 [0.07-0.55]	0.25 [0.12-0.91]	0.37 [0.18-1.23]	0.03 [0.02-0.05]	0.05 [0.03-0.10]	0.07 [0.04-0.13]
GE - Catheter	PA4D	675	0.16 [0.07-0.54]	0.25 [0.12-0.88]	0.37 [0.18-1.20]	0.03 [0.02-0.05]	0.05 [0.03-0.10]	0.07 [0.04-0.13]

Note. *The Clarius (HH) and GE (PA) results were obtained from independent experimental datasets and are therefore reported with their respective Empty conditions. Nominal n corresponds to 27 positions x 25 holes. RMS, P95, and maximum deviations are summarized across valid locations as median [Q1-Q3].*
